# Introducing the Climate Change Special Issue

**DOI:** 10.5811/westjem.66424

**Published:** 2026-09-24

**Authors:** Gary Gaddis, Shahram Lotfipour

**Affiliations:** University of California, Irvine School of Medicine, Department of Emergency Medicine, Orange, California

The *Western Journal of Emergency Medicine* (*West*JEM*)* is pleased to present its Climate Change Special Issue. These 23 manuscripts explore how choices in emergency department (ED) operations and processes affect climate change drivers, including greenhouse gas emissions. This special issue grew out of my suggestion to Editor-in-Chief Mark Langdorf, MD, MHPE, that the journal focus on climate issues, similar to its periodic special issues on medical education. Several factors prompted this suggestion:

- In the United States, the healthcare industry contributes about 8.5% of all greenhouse gases produced in our economy.^1^
- Choices in emergency care delivery directly influence greenhouse gas production. When I first proposed this issue, I had recently served as Decision Editor for a *West*JEM manuscript quantifying how the use of reusable, sterilizable oximeter probes in the ED could favorably reduce these emissions.^2^
- When the journal issued its “Call for Papers,” the American Medical Association (AMA) had already adopted at least 12 policies addressing climate change.^3^ These policies include the following:
  ○ Policy D-135.966 (last modified in 2024) declares climate change to be a public health crisis. It further states, the AMA “will protect patients by advocating for policies that:
    ▪ Limit global warming to no more than 1.5 degrees Celsius
    ▪ Reduce US greenhouse gas emissions aimed at a 50 percent reduction in emissions by 2030 and carbon neutrality by 2050.”
  ○ Policy D-440-912 (last modified in 2023) advocates for a “net zero” carbon society by 2050.
- Increased implementation of “telemedicine” since the onset of the COVID-19 pandemic has had a small but measurable and salutatory effect upon the healthcare industry’s net output of greenhouse gases.

Subsequently, at the June 2026 Annual Meeting of the House of Delegates of the AMA, Resolution 404-A-26 was adopted, adding to the impetus to make climate-conscious resources more available to clinicians. Its “Resolved” clauses include the following:

- RESOLVED, that our American Medical Association support compiling and maintaining a resource for US physicians, focused on education physicians and other healthcare professionals about the health impacts of climate change and the role of the healthcare sector in contributing to greenhouse gas emissions; and be it further
- RESOLVED, that our AMA support providing practical, evidence-based recommendations for reducing the environmental footprint of clinical practices and healthcare systems; and be it further
- RESOLVED, that our AMA support offering resources and tools to support physicians in advocating for environmentally sustainable policies and practices within their organization and communities; and be it further
- RESOLVED, that our AMA facilitate collaboration and sharing of best practices among healthcare professionals and institutions committed to addressing climate change and promoting sustainability.

We hope this issue will support the AMA’s goals within our emergency medicine community. Mitigating healthcare industry greenhouse gas emissions to eventually achieve carbon neutrality will require multiple strategies rather than a single “magic bullet.” However, important incremental steps are already uuderway. For example, the University of California, Irvine, recently opened the first “all-electric” hospital in the United States.^4^ Substantial, and perhaps surprising, progress is also occurring in emergency services delivery:

- Great Britain’s National Health Service (NHS) committed to a “net-zero” carbon footprint by 2040, followed by a net-zero target for its wider supply chain and influenced emissions by 2045.^6^ As part of this initiative, the NHS is systematically phasing out emergency medical service (EMS) vehicles powered by fossil fuels and replacing decommissioned units with zero-emission models.^5^
  ○ While both Great Britain and the US have extensive rural areas, the British advantage lies in the fact that the NHS operates all ambulance services, compared with the “patchwork” of agencies operating in the US.
- Nonetheless, this EMS vehicle goal is being rapidly met within some British EMS systems, surprisingly in areas that are not densely populated metropolitan centers like Central London.
  ○ The NHS Hillingdon Hospitals, located west of London and north of Heathrow Airport, have already transitioned their EMS vehicles to 100% electrically powered.^6^
  ○ In Yorkshire, a largely rural northern area of the United Kingdom that also includes the cities of York and Leeds, 35 of a planned 108 renewably powered ambulances are already in service.^7^

*West*JEM’s attention to climate change is not unique. As a regular reader of the *Journal of the American Medical Association*, I know that as of June 2026, *JAMA* had published at least 20 climate-related articles and news items. So, in closing, when viewed from the perspective of 2030 or even 2050, this “Special Issue” will hopefully be viewed as a vital first step taken by our emergency medicine community toward reducing our collective greenhouse gas production. As the proverb states, “A journey of 1000 miles begins with but a single step.”

In that spirit, we present our Special Climate Change Issue.
